# Outcome Selection for Research Studies in Movement Disorders

**DOI:** 10.1002/mdc3.14087

**Published:** 2024-06-03

**Authors:** Susan H. Fox

**Affiliations:** ^1^ University of Toronto, Movement Disorder Clinic, Edmond J Safra Program in Parkinson Disease, Toronto Western Hospital, University Health Network Toronto ON Canada

**Keywords:** clinical trials, outcome measures, primary outcome measures, secondary outcome measures

## An Understanding of Disease Semiology is Key

Choosing the correct outcome measure for any clinical research study in the field of movement disorders depends on the disease being studied and the research question being asked.

The clinical features of the movement disorder will impact *which* outcome measure; *when* it will be measured and *how* it will be measured. For example, clinical studies investigating Parkinson's disease (PD) requires knowledge of the myriad of motor and non‐motor features of the disease, not only in choosing the correct scale but also the potential impact of other disease features on the proposed outcome measures. The heterogeneity of PD will also affect the type of outcome measure if focussed on one component of the disease for example, tremor versus non‐tremor dominant. Outcomes used in dystonia need to be tailored to the body distribution of dystonia such as focal or generalized, as well as other components such as pain and tremor. Some movement disorders are progressive and worsen over time, such that the outcome measure used will impact the trial design. Thus, in early untreated PD, studies using a motor score (eg, MDS‐UPDRS III) as a surrogate for “disease progression” when investigating a potential neuroprotective agent, could require a trial to last months to years as the change in MDS‐UPDRS III in the early stages of disease is small and non‐linear (eg, 3–4 points/year).^1^ While later in the disease, the same outcome, MDS UPDRS III, is a measure of symptomatic effects of an intervention and a shorter duration of trial is sufficient. Likewise the effect of symptomatic medications needs to be incorporated into any study investigating potential disease‐modification in PD when the MDS‐UPDRS is used as an outcome measure. Other challenges to take into consideration include knowledge of the natural history of the movement disorder such as fluctuations in symptoms. This may occur with disorders such as Tics and Tourette's syndrome, and truly paroxysmal movement disorders that may impact the timing and type of outcome measure used. Likewise, fluctuations or changes in symptoms due to symptomatic therapy in PD with motor and non‐motor fluctuations is also a major variable to consider when choosing an outcome measure. The natural history of other symptoms in PD may also fluctuate, including psychosis, dyskinesia and many non motor issues that may impact type and timing of outcome measures.^2^ Thus knowledge of the seminology is key to designing and incorporating the correct outcome measures.

## The Research Question and Hypothesis Being Tested Will Determine the Outcome Measure

In the traditional stages of translational clinical research, an intervention that shows benefit in preclinical studies will next be evaluated in healthy participants for safety and tolerability (phase I trial). The “first‐into‐patient” studies (phase Ib/II trials) are to determine *if* an intervention works and usually requires an objective (observer‐rated) measurement. Phase III studies double blind randomized controlled trials (DBRCTs) are to determine *if and how well* an intervention works, and thus outcome measures usually include a mixture of objective and subjective measures including patient‐reported outcomes. Hypothesis‐generating clinical research may also lead on from a clinical observation (see viewpoint by Dr A. Fasano). The research question then will usually be to corroborate the finding such as point prevalence of the observation could be carried out where the outcome measure is a qualitative survey.

## Four Principles of Choosing an Outcome Measure for a Clinical Research Study

1. The Intervention will *change* the outcome measure. There needs to be a sufficient measurable change to enable appropriate statistical analysis to be performed. For example, an appropriate outcome measure for assessing the benefit of an intervention on motor fluctuations in PD could include a patient‐reported diary^3^ documenting duration (time) over the waking day when ON or OFF medication.
2. The change in the outcome measure is *relevant*. The change (positive or negative) needs to have clinical relevance or be meaningful to all stakeholders, including the patient, caregiver, health care providers and society, if relevant. Thus although there is a measurable, or statistically significant change in the outcome, the change needs to be clinically relevant. Determining Minimally Clinically Relevant Difference (MCID) has been performed for some movement disorder scales (see below)
3. The outcome measure is *easily measured*. The best outcome measure is one that can be determined in a timely manner, with least disruption to the participant. Biological markers such as CSF are obviously more invasive as an outcome than a blood sample. A rating scale also needs to be easily administered, and time efficient for both rater and participant. For example, comparisons of rating scales for measuring levodopa‐induced dyskinesia have shown time to administer these scales varies from 2 to 20 min.^4^
4. The outcome measure is *reliable and validated*. When repeatedly using the chosen outcome measure the same results should be obtained with good inter‐ and intra‐rater reliability, if all other methodological conditions are kept constant. Clinimetric testing evaluation of consistency, validity and responsiveness of most commonly used scales used in movements disorders have been performed.^5^

## Objective Versus Subjective Outcome Measures

Objective outcome measures used in clinical research studies of movement disorders range from population‐based health record database metrics, to observer–rated clinical scales or less commonly a laboratory biomarker. Epidemiological studies are the most objective type of research due to the anonymized nature of the data. Outcome measures used in large population studies consist of well‐defined metrics such as mortality, health‐care utilization, medication use etc.^6^

For clinical trials assessing the benefit of an intervention, then use of an objective outcome measure is less prone to bias and minimizes placebo (false positive) or nocebo (false negative) effects that are high in some movement disorders such as PD.^7^ The most common objective outcome measures (predominantly rating scales) in movement disorders are available on the MDS website^8^ and reviewed in viewpoint by Dr Mayela. Knowing the MCID of an objective scale can help determine how long an intervention should be given in a trial (ie, time for the expected change in MCID to occur with the intended intervention) and helps determine the frequency of outcome measures needed in the trial. Several scales used in movement disorders have had MCID values determined; including for the MDS‐UPDRS in early and advanced PD^9^, ^10^; Unified Dyskinesia Rating Scale (UDyRS)^11^ and King's PD Pain Scale^12^; generalized dystonia using the Burke‐Fahn‐Marsden Dystonia scale^13^; Tardive dyskinesia in the Abnormal Involuntary Movement Scale^14^; and for early and advanced stages using Unified Huntington's Disease Rating Scale (UHDRS).^15^

In the field of movement disorders, there are currently no validated biomarkers for measuring disease progression. In PD, proteomics, metabolomics and imaging, are potentially used for diagnosis, but most have not yet shown validity in tracking disease progression or change in response to a symptomatic intervention.^16^, ^17^ As an alternative in PD, clinically‐relevant “milestones” have been suggested as well‐defined objective outcomes in evaluating baseline factors that may predict speed of disease progression including time to frequent falls, dementia and time to residential care^18^; however such studies require long‐term follow‐up as the outcome measure reflects change over several years. More recently, a similar approach showed that clinically‐relevant milestones such as functional dependence; walking and balance changed in over 50% of a cohort of early PD subjects by 5 years suggesting these potentially more relevant outcomes could be useful measures of disease progression at earlier stage of PD.^19^ The other advantage of a milestone outcome marker is that it takes into account the heterogeneity of the disease.

Objective measures of gait parameters, such as speed, distance and stride length, have been used successfully in some PD trials.^20^ Other timed measures used in PD studies include the Purdue pegboard test which is a measure of upper extremity bradykinesia.^21^ A recent group proposed using a combination of these outcome measures with the UPDRS III motor subscale, to increase sensitivity to change in subjects with early PD and thus reduce number of subjects required to be recruited in a trial by ~60%.^22^ Wearable devices or mobile health technologies^23^ as objective outcome measures for movement disorders continue to increase in use, especially in PD and tremor. However, there remain challenges with understanding the relevance of the “big‐data” generated and ongoing validation is required to determine clinical relevance and thus benefit as an outcome for clinical research.^24^, ^25^ The usefulness and validity of measuring non‐motor symptoms in PD with such devices seems to be even more challenging.^26^

Subjective outcomes are usually patient‐reported measures that include symptom impact on daily activities and disability, health‐related quality of life and satisfaction with treatment. Many disease‐specific validated rating scales used in movement disorders incorporate a mixture of objective and subjective ratings for example, the MDS‐UPDRS parts I–IV for PD; Toronto Western Spasmodic Torticollis Rating Scale (TWSTRS) parts I–III for cervical dystonia, and the Tremor Rating Scale. Use of part of the whole scale, ie subscale measuring one aspect of the disease, is often used as a valid outcome measure, although it is unusual to choose a purely subjective outcome measure as the primary outcome for a clinical trial. In many regulatory‐approval phase III RCTs for symptomatic therapies in PD, a combination of MDS‐UPDRS II and III has been used as the primary outcome measure; however the true validity of such composite scores is unclear due to the mixture of objective with subjective outcomes.^27^ In many phase III RCTs of symptomatic therapies for PD, patient‐reported diaries documenting symptoms are a frequent endpoint. These diaries incorporate a mixture of objective (time) with subjective (patient‐reported) outcomes. Thus, reporting the daily time spent in “ON” with or without dyskinesia; or “OFF” is a validated method and has been incorporated into many, now approved, interventions for PD. Knowledge of the semiology of levodopa‐induce dyskinesia means that many people with PD are unaware that they have dyskinesia and may only report the presence of dyskinesia if it becomes troublesome.^28^ Thus, defining the ON as “ON‐ with or without troublesome dyskinesia” reflects the patients perceived level of disability associated with dyskinesia, as opposed to an objective rating of the amount of dyskinesia.^3^ Non‐disease specific subjective outcomes such as Clinical Global Impression of change (CGI) are also often used in movement disorder studies due to patient‐centric nature and ease of administration.^29^

To better standardize patient‐reported outcomes for clinical studies, there are global efforts to harmonize scales through the International Consortium for Health Outcomes Measurement (ICHOM).^30^ The aim is to have outcomes for a disease that are meaningful for patients, cost‐effective and that are universally accepted and can be compared across countries. There is an ICHOM recommendation for PD that includes scale of motor and non‐motor symptoms, ability to work, PD‐related health status, and hospital admissions.^31^ The US FDA has also developed a Clinical Outcome Assessment compendium^32^ that is a guide for pharmaceutical industry studies to design more patient‐focussed trials and to date, includes suggested scales for trials in blepharospasm, cervical dystonia, HD, PD and restless‐legs syndrome. Although these are proposed for regulatory‐required clinical trials, such suggested scales maybe useful for investigator‐initiated trials. For example, a recent expert opinion review has suggested a range of outcome measures that includes a mixture of objective scales plus biomarkers, wearable, plus subjective quality of life and milestones as a benchmark for accelerating future trials of potential interventions in PD.^33^

## Primary and Secondary Outcome Measures

Within the CONSORT guidelines for reporting clinical trials,^34^ primary and secondary outcomes must be pre‐specified and clearly defined as to how, and when, they were assessed. The primary outcome measure is determined from the research question and should reflect the hypothesis being tested. A single primary outcome measure is usually defined and is then used to determine the number of subjects required for statistical validity. Secondary outcomes will vary in number and depend on the nature of the study, but include other measures of the movement disorder being evaluated for example, any PD study of anti‐dyskinetic or non‐motor symptom requires a measure of PD motor disability. Effects on other aspects of the disease should be included that will enable tolerability of the interventions and in most movement disorders typically include outcome measures of sleepiness, mood (depression and anxiety rating scales) and suicide risk.

## How and when to Measure Outcomes

The primary outcome measurement needs to be administered in an unbiased manner and ideally by a rater unaware or “blind” to the intervention. Secondary outcome measures can be measured by a treating or non‐blinded rater. The timing of assessment includes ensuring all variables that may affect the outcome measure on the day/days are taken into consideration for example, natural fluctuation of the disease; time of day of evaluation, effect of concomitant medications such as levodopa in PD (on vs. off) or effect of botulinum toxin in dystonia etc.

The duration of a study will also be determined by the outcome measure chosen. In most movement disorders, due to the underlying pathophysiology, the potential change in a motor symptom and most non‐motor symptoms are slow and would be expected to take days to weeks to be apparent. As noted above, knowledge of the MCID will assist in timing of outcome measures. Practical issues also need to be considered including time‐efficiency for participants in studies, and financial costs of longer duration trials. Thus for most RCT phase III trials of a potential symptomatic therapy in many movement disorders including PD, dystonia, tremor, 12 weeks is often used as a reasonable time frame. Shorter duration studies may be possible in some movement disorders. An intervention for levodopa‐induced dyskinesia in PD can potentially be evaluated in an acute levodopa challenge paradigm when a drug is administered acutely or for a few days and then effect on reducing dyskinesia measured with UDyRS III used as a primary outcome. Orthostatic hypotension therapies in PD potentially could be evaluated over a few days as an outcome could be change in postural BP; with then a potential longer‐term trial to determine benefit of Patient reported outcomes such as symptomatic dizziness or falls.

## Conclusion

Choosing the required outcome measures for clinical studies needs to be a balance between doing clinical research to International Council for Harmonization of Technical Requirements for Pharmaceuticals for Human Use (ICH) Good Clinical Practice (GCP) standards^35^ with being pragmatic, realistic, clinically relevant, and financially viable. Outcome measures used in regulatory‐required clinical trials are usually beyond the scope of individual clinicians/Principal Investigators (PIs) as the outcomes measures are decided usually well before a site is asked to participate in a trial. However, as experts in the field of movement disorders, critical feedback can, and should, be provided if desired outcome measures are deemed challenging to measure. This dialogue between PIs, industry and regulatory authorities helps and can potentially lead to better‐designed, patient focussed clinical trials. For investigator‐initiated studies, the number of outcome measures should be kept to the minimum required to test the hypothesis. The more outcomes imposed on study subjects then the harder, longer and more expensive the study becomes with little extra gain. Always remember the patient's voice in designing clinical studies. The opinions from patient‐advisory boards and patient‐partners are now regularly utilized and extremely helpful in designing research.^36^ The patient ultimately wants a cure or treatment for their disease as soon as possible, and by choosing outcomes measures that are going to expedite this end as quickly as possible is beneficial for all (Video 1).

**Video 1:** 2024 MDCP conference recording.

## Author Roles

Manuscript Preparation: A. Writing of the first draft, B. Review and Critique.

S.F.: A, B, C

## Disclosures

**Ethical Compliance Statement:** The authors confirm that no institutional review board/patient consent was required for this work. I confirm that I have read the Journals’ position on issues involved in ethical publication and affirm that this work is consistent with those guidelines.

**Funding Sources and Conflict of Interest:** No specific funding was received for this work. The author declares that there are no conflicts of interest relevant to this work.

**Financial Disclosures for Previous 12 Months:** Clinic support from the Edmond J. Safra Foundation for Parkinson Research; Parkinson Foundation and the Toronto Western and General Foundation. Research Funding from Michael J. Fox Foundation for Parkinson Research, NIH (Dystonia Coalition); Parkinson Canada; Weston Foundation. Honoraria from the International Parkinson and *Movement Disorder* Society. Site PI for Clinical Trials for Alexion, Biotie, Consultancy/Speaker fees from Abbvie, Lundbeck, Sunovion; Royalties from Oxford University Press.
